# Effects of rosuvastatin on ADMA, rhokinase, NADPH oxidase, caveolin-1, hsp 90 and NFkB levels in a rat model of myocardial ischaemia–reperfusion

**DOI:** 10.5830/CVJA-2014-038

**Published:** 2014

**Authors:** Oktay Burma, Ayhan Uysal, Elif Onat, Engin Sahna, Necip Ilhan, Deniz Erol, Mete Ozcan

**Affiliations:** Department of Cardiovascular Surgery, Faculty of Medicine, University of Firat, Elazig, Turkey; Department of Cardiovascular Surgery, Faculty of Medicine, University of Firat, Elazig, Turkey; Department of Pharmacology, Faculty of Medicine, University of Firat, Elazig, Turkey; Department of Pharmacology, Faculty of Medicine, University of Firat, Elazig, Turkey; Department of Biochemistry, Faculty of Medicine, University of Firat, Elazig, Turkey; Department of Medical Genetics, Faculty of Medicine, University of Firat, Elazig, Turkey; Department of Biophysics, Faculty of Medicine, University of Firat, Elazig, Turkey

**Keywords:** ischaemia–reperfusion, rosuvastatin, oxidative stress, ADMA, hsp 90, caveolin-1, NFkB, rhokinase, NADPH oxidase

## Abstract

**Aim:**

Endothelial dysfunction, oxidative stress and inflammation are among the most important mechanisms of ischaemia–reperfusion (I/R) injury. Besides their cholesterol-lowering effects, statins are known to provide protection against myocardial dysfunction and vascular endothelial injury via nitric oxide-dependent mechanisms. The aim of this study was to investigate the effects of rosuvastatin on certain intermediates involved in the generation of nitric oxide (asymmetrical dimethyl arginin, ADMA, caveolin-1 and hsp 90), oxidative stress (rhokinase, NADPH oxidase) and inflammation (NFkB), using an *in vivo* model of myocardial infarction in the rat.

**Methods:**

Adult male Sprague Dawley rats were divided into three groups (control, I/R and I/R after 15 days of rosuvastatin administration). Reperfusion was applied for 120 min following left anterior descending coronary artery ischaemia for 30 min. Caveolin-1, hsp 90 and NFkB levels were evaluated with the quantitative reverse transcriptase polymerase chain reaction (qRT-PCR) and ADMA, rhokinase and NADPH oxidase levels were evaluated with ELISA.

**Results:**

While NFkB and hsp 90 levels were higher in the I/R group, their levels were significantly lower in the rosuvastatin group. While ADMA and NADPH oxidase levels significantly increased with I/R, they were lower in the rosuvastatin-treated group, but not statistically significant. Rhokinase levels were significantly lower in the rosuvastatin group. Caveolin-1 levels were not different between the groups.

**Conclusion:**

Our results suggest that ADMA, rhokinase, NADPH oxidase, hsp 90 and NFkB could facilitate I/R injury, and rosuvastatin significantly reduced levels of these parameters. These results indicate that rosuvastatin may have a protective role in I/R injury via mechanisms targeting inflammation, endothelial dysfunction and oxidative stress.

## Abstract

Ischaemic heart disease remains among the major causes of morbidity and mortality worldwide. The most common form is reduction in blood flow in the coronary arteries supplying blood to the myocardium due to atherosclerotic plaques or vasospasm.^1^ After ischaemia, reperfusion of the tissue is of great importance for maintenance of the viability of the ischaemic tissue. However reperfusion may paradoxically lead to some morphological changes, enzyme destruction and even death of the still-viable tissue that may be rescued.^2^

Ischaemia–reperfusion (I/R) injury is the mainstay of myocardial infarction, cerebral ischaemia, stroke, haemorrhagic shock and surgical interventions such as organ transplantation, cardiac surgery, coronary angioplasty and thrombolytic treatment-related pathophysiology.^3^ Endothelial dysfunction, oxidative stress and inflammation are among the most common mechanisms of I/R injury.^4^,^5^

Asymmetrical dimethyl arginine (ADMA) is an endogenous nitric oxide synthase (eNOS) inhibitor. Its importance is becoming more recognised and further studies are required to determine its use in clinical diagnosis. Available evidence indicates that oxidative stress leads to changes in the activity of enzymes involved in the production and degradation of ADMA.^4^,^5^ High levels of ADMA and low levels of nitric oxide (NO) in the coronary arteries of patients with vasospastic angina have been reported.^6^

In the cardiovascular system, NADPH oxidase accounts for the production of reactive oxygen species (ROS), which is produced not only during I/R injury but also under physiological conditions.^7^ The pro-oxidative NADPH oxidase is present in the plasma membranes of neutrophils, which are an important source of free radical formation and I/R injury.^8^ Additionally, the rhokinase pathway, which has an important role in regulation of vascular smooth muscle tone, has been shown to be involved in I/R injury, thus making its inhibition a potential target for limiting I/R injury.^9^

It has been reported that inflammatory NFkB expression increased in the I/R-related infarct area; inflammation was suppressed when NFkB expression was inhibited, and cardiac preservation was provided.^10^ In this context, caveolin-1 was shown to regulate eNOS activation consistently with other signalling molecules such as hsp 90.^11^ Interaction of hsp 90 with eNOS increases eNOS activity, and consequently, NO production increases.^12^,^13^ Myocardial caveolin-1 content is reported to decrease following ischaemia–reperfusion.^14^ Caveolin-1 deficiency was noted to aggravate cardiac dysfunction and reduce the survival rate in mice that had experienced myocardial infarction (MI).^15^

Rosuvastatin is a synthetic hydrophilic statin widely used in the treatment of dyslipidaemia, as it increases levels of highdensity lipoprotein (HDL) cholesterol, and reduces low-density lipoprotein (LDL) cholesterol and triglyceride levels. Statins have been reported to have anti-inflammatory, antiproliferative, antithrombotic, anti-atherogenic and antihypertensive effects in addition to their cholesterol-lowering effects.^8^,^16^-^18^ Recent studies indicate that rosuvastatin decreases levels of ADMA in hypercholesterolaemia,^19^ levels of caveolin,^20^ and also NFkB levels^21^ in subaracnoid bleeding.

To our knowledge, the effects of rosuvastatin on ADMA, rhokinase, caveolin-1, hsp 90 and NFkB levels are not known in cardiac I/R injury. In this study, we aimed to investigate the influence of rosuvastatin on oxidative stress-related rhokinase, NADPH oxidase, ADMA, caveolin-1 and hsp 90 levels in a rat model of I/R injury.

## Methods

Male Sprague Dawley rats weighing 250–300 g were kept in a quiet, temperature- (21 ± 2°C) and humidity- (60 ± 5%) controlled room in which a 12-hour light–dark cycle was maintained. All experiments were performed between 9:00 and 17:00.

The rats were divided into three groups: control (sham), I/R + vehicle (physiological saline) and I/R + rosuvastatin. Vehicle or rosuvastatin (10 mg/kg) were administered in the afternoon (17:00) by intraperitoneal injection for 15 days before ischaemia. I/R protocols were performed in the morning (08:00–12:00).

Measurement of myocardial tissue rhokinase, NADPH oxidase, caveolin-1, hsp 90, NFkB and ADMA levels were performed in seven animals in each group. Rosuvastatin (Abdi Ibrahim Pharmaceutical Co, Istanbul, Turkey) was dissolved in physiological saline.

All experiments in this study were performed in accordance with the guidelines for animal research from the National Institutes of Health and were approved by the local committee on animal research (FUHADYEK -13.06.2012-76).

## Ischaemia–reperfusion procedure

Rats were anesthetised with urethane (1.2–1.4 g/kg) administered intraperitoneally. The jugular vein and trachea were cannulated for drug administration and artificial respiration, respectively. Systemic blood pressure (BP) was monitored via the carotid artery with a Harvard model 50-8952 transducer (Harvard Apparatus Inc, Massachusetts, USA) and displayed on a Harvard Universal pen recorder (Harvard Apparatus, Inc, Massachusetts, USA) together with a standard 12-lead ECG.

The chest was opened via a left thoracotomy, followed by sectioning the fourth and fifth ribs, about 2 mm to the left of the sternum. Positive-pressure artificial respiration was started immediately with room air, using a volume of 1.5 ml/100 g body weight at a rate 60 beats/min to maintain normal pCO_2_, pO_2_ and pH parameters.

After the pericardium was incised, the heart was exteriorised by gentle pressure on the outside of the rib cage. A 6/0 silk suture attached to a 10-mm micropoint reverse-cutting needle was quickly placed under the left anterior descending coronary artery. The heart was then carefully replaced in the chest, and the animal was allowed to recover for 20 min. Any animal in which this procedure produced arrhythmias or a sustained decrease in mean arterial BP to less than 70 mmHg was discarded.

A small plastic snare was threaded through the ligature and placed in contact with the heart. The artery could then be occluded by applying tension to the ligature, and reperfusion was achieved by releasing the tension. At the end of the experimental period, left ventricle myocardial samples, distal to the left main coronary artery occlusion, were collected for analysis and analysed within one month.

## Quantitative real-time polymerase chain reaction analysis (qRT-PCR)

Tissue samples were immersed in RNAlater. After overnight saturation with RNAlater, the tissues were stored at –80°C. All protocols were performed according to the manufacturer’s instructions. Total RNA was extracted from rat heart tissues using TRizol reagent (Invitrogen, Carlsbad, USA).

To carry out the PCR assay, total RNA from the heart samples in each experimental group was pooled (3 μg total). cDNA from the pooled samples was synthesised using a highcapacity RNA-to-cDNA kit (Invitrogen, Carlsbad, USA). Relative expression levels of mRNA were determined using a 7500 fast real-time PCR (PE Biosystems, Foster City, CA, USA) with Taq Man master mix and rat-specific assays for NFkB, caveolin-1, hsp 90 and GAPDH genes. The relative abundance of mRNA was calculated after normalisation to GAPDH.

Triplicate assays were performed. PCR reactions were performed after heating to 50°C for 2 min followed by 40 cycles of denaturation at 95°C for 10 min, 95°C for 15 sec and 60°C for 1 min. ADMA, rhokinase and NADPH oxidase levels were evaluated with ELISA.

## Statistical analysis

Data are expressed as arithmetic means ± SEM. When *p* < 0.05, the difference was considered to be statistically significant. Normality of the distribution within the groups was evaluated with the Shapiro–Wilk test. Multiple comparisons between the experimental groups were performed by one-way analysis of variance with the Tukey post hoc test.

## Results

**Fig. 1.**
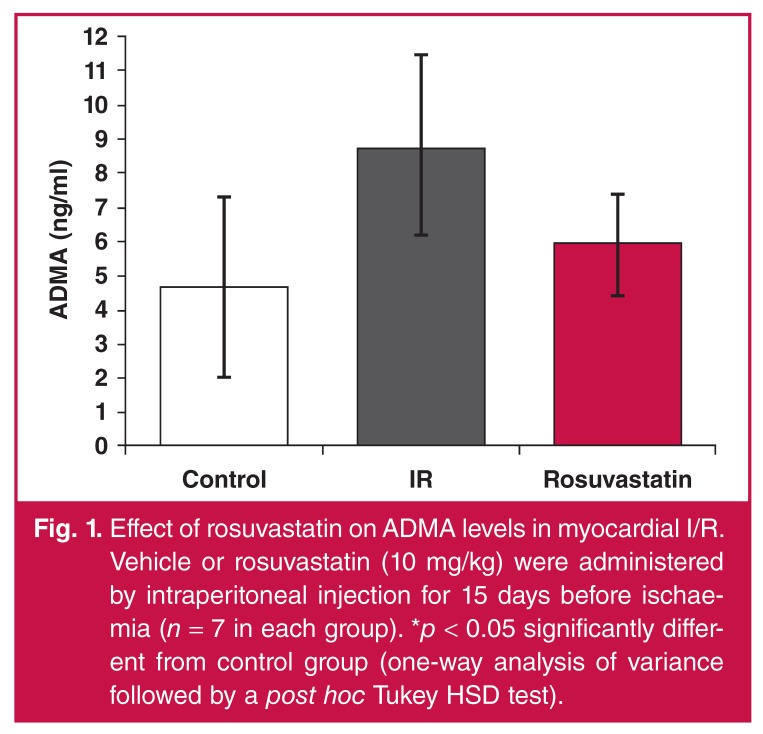
Effect of rosuvastatin on ADMA levels in myocardial I/R. Vehicle or rosuvastatin (10 mg/kg) were administered by intraperitoneal injection for 15 days before ischaemia (*n* = 7 in each group). *p < 0.05 significantly different from control group (one-way analysis of variance followed by a post hoc Tukey HSD test).

I/R caused a significant increase in ADMA levels. This increase was limited although not statistically significantly attenuated in the rosuvastatin group (Fig. 1).

**Fig. 2.**
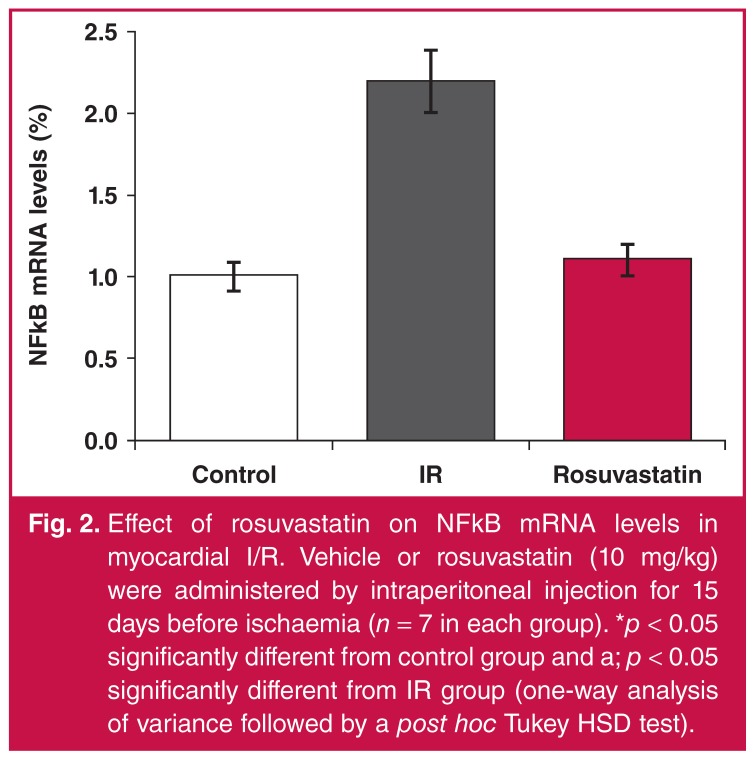
Effect of rosuvastatin on NFkB mRNA levels in myocardial I/R. Vehicle or rosuvastatin (10 mg/kg) were administered by intraperitoneal injection for 15 days before ischaemia (*n* = 7 in each group). *p < 0.05 significantly different from control group and a; *p* < 0.05 significantly different from IR group (one-way analysis of variance followed by a post hoc Tukey HSD test).

**Fig. 3.**
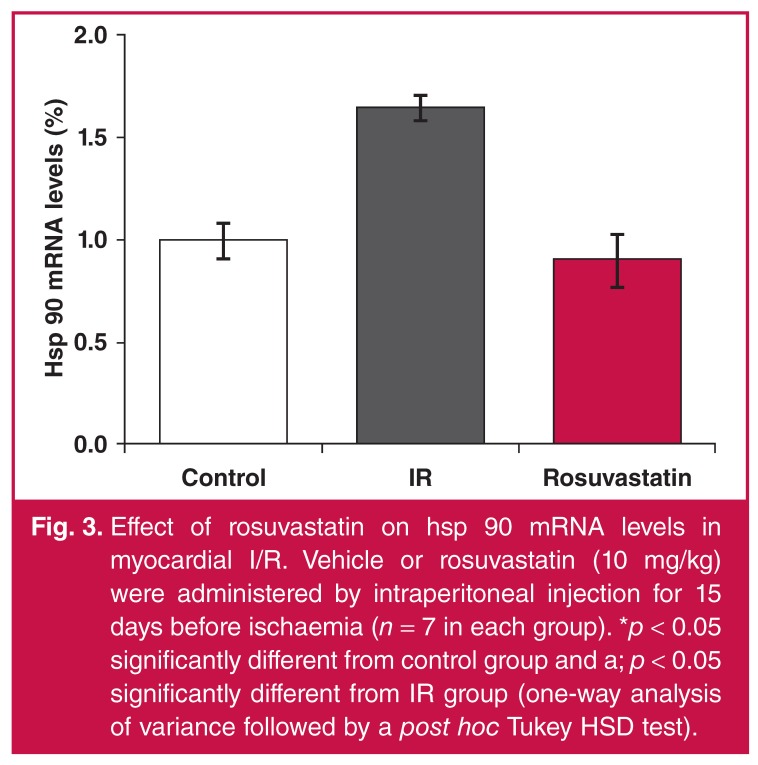
Effect of rosuvastatin on hsp 90 mRNA levels in myocardial I/R. Vehicle or rosuvastatin (10 mg/kg) were administered by intraperitoneal injection for 15 days before ischaemia (*n* = 7 in each group). *p < 0.05 significantly different from control group and a; *p* < 0.05 significantly different from IR group (one-way analysis of variance followed by a post hoc Tukey HSD test).

**Fig. 4.**
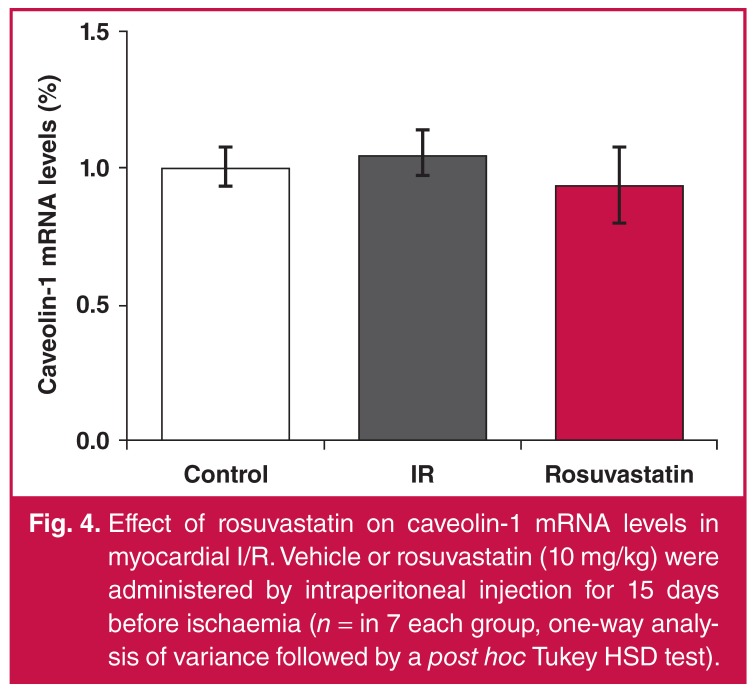
Effect of rosuvastatin on caveolin-1 mRNA levels in myocardial I/R. Vehicle or rosuvastatin (10 mg/kg) were administered by intraperitoneal injection for 15 days before ischaemia (*n* = in 7 each group, one-way analysis of variance followed by a *post hoc* Tukey HSD test).

While NFkB levels increased 2.2-fold with I/R, they significantly decreased in the rosuvastatin-treated group (Fig. 2). While hsp 90 levels increased 1.6-fold in the I/R group, they decreased significantly in the rosuvastatin group and returned to control values (Fig. 3). There was no significant difference between groups in terms of caveolin-1 levels (Fig. 4).

**Fig. 5.**
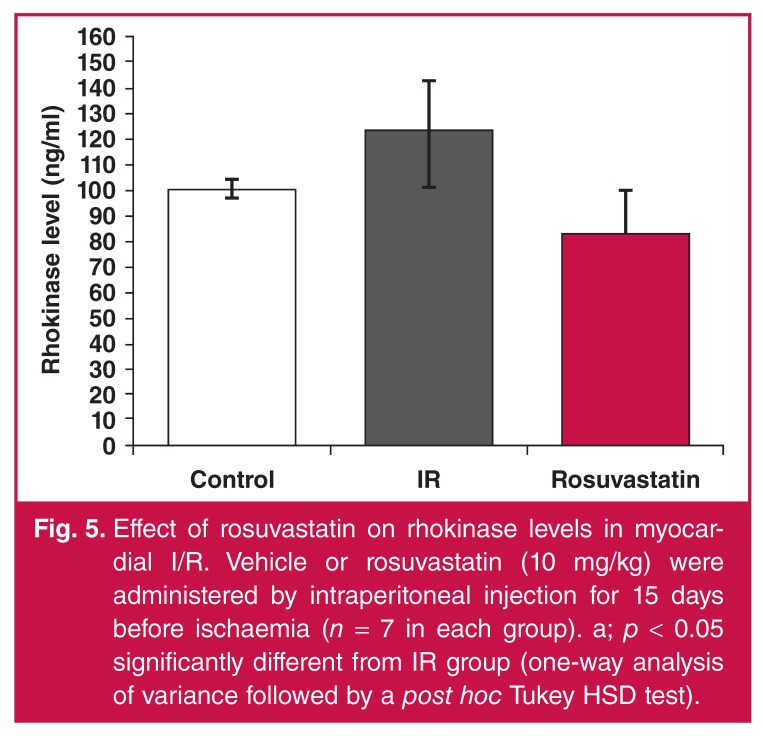
Effect of rosuvastatin on rhokinase levels in myocardial I/R. Vehicle or rosuvastatin (10 mg/kg) were administered by intraperitoneal injection for 15 days before ischaemia (*n* = 7 in each group). a; *p* < 0.05 significantly different from IR group (one-way analysis of variance followed by a post hoc Tukey HSD test).

**Fig. 6.**
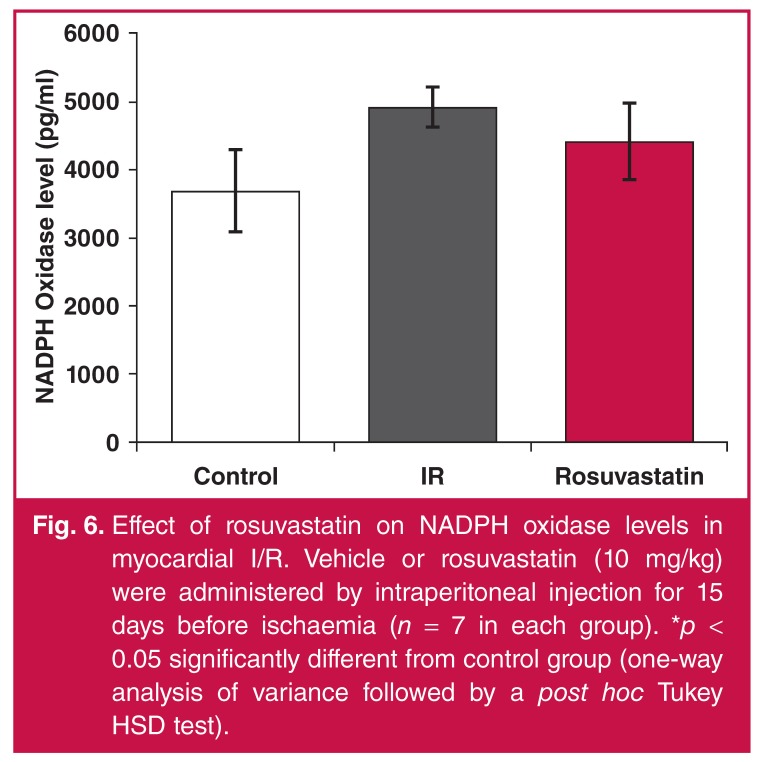
Effect of rosuvastatin on NADPH oxidase levels in myocardial I/R. Vehicle or rosuvastatin (10 mg/kg) were administered by intraperitoneal injection for 15 days before ischaemia (*n* = 7 in each group). *p < 0.05 significantly different from control group (one-way analysis of variance followed by a post hoc Tukey HSD test).

NADPH oxidase levels significantly increased with I/R, and a limited but statistically significant attenuation was observed in only the rosuvastatin group (Fig. 5). Similarly, rosuvastatin treatment was able to significantly attenuate the increase in rhokinase levels (Fig. 6).

## Discussion

Results from this study show that 15-day intraperitoneally injected rosuvastatin was able to decrease the myocardial injury caused by I/R. Ischaemia–reperfusion itself increased tissue NFkB, hsp 90, ADMA, and NADPH levels without significantly changing caveolin-1 levels. According to our results, rosuvastatin inhibited changes in levels of NFkB, hsp 90, rhokinase, ADMA and NADPH oxidase but not caveolin-1 levels in rat cardiac tissue with induced myocardial I/R.

The beneficial effects of statins have been shown in cardiovascular diseases, including acute coronary syndromes.^8^,^18^,^22^ It was reported that rosuvastatin may have protective effects in I/R injury and these effects could have been mediated by immunomodulatory and anti-inflammatory effects.^7^,^17^,^23^ Kuhn *et al.*^24^ showed that myocardial function improved with rosuvastatin administration for seven days prior to cardiopulmonary bypass surgery. The protective effects of rosuvastatin regarding antioxidant and anti-inflammatory properties have also been reported in brain I/R models.^25^

ADMA is an endogenous NOS inhibitor competing with L-arginin to bind to NO. The plasma ADMA level was reported to be elevated in coronary artery disease and it is seen to be a risk factor with a worse clinical outcome for percutaneous coronary interventions.^26^-^28^ Studies have consistently indicated that cardiac I/R caused elevation in levels of serum ADMA^29^ and myocardial tissue ADMA.^30^ In our study, tissue ADMA levels were elevated with I/R, which was reduced in the rosuvastatin group.

Elevated NADPH levels lead to elevation in ROS levels and decreased bioavailability.^7^ NADPH oxidase activity was reported to increase in the heart with I/R. NADPH oxidase was shown to be related to platelet activation and thrombus formation in I/R.^8^ In our study, the NADPH oxidase level increased with I/R and this elevation decreased with rosuvastatin administration.

Pignatelli *et al.*^8^ demonstrated that rosuvastatin caused antiplatelet activity independent of its lipid-lowering effect and this was related to its effect of reducing NADPH oxidase levels. In the same study, rosuvastatin was shown to reduce oxidative stress by reducing NADPH oxidase levels, upregulating antioxidant enzymatic defence mechanisms and inhibiting hydrogen peroxide-mediated DNA damage.

Hsp 90 is a cytoprotective protein chaperone that participates in mitochondrial import of a number of proteins. It was shown to increase I/R-related necrotic cell death when blocked pharmacologically.^31^ Hsps are reported to be protective by being upregulated in the case of increased oxidative stress.^32^ Under our experimental conditions, the hsp 90 level was seen to increase as a protective mechanism during I/R. We believe that the expected increase in hsp 90 levels would not have been seen together with the decrease in injury due to the positive effects of rosuvastatin on the other parameters.

Caveolin-1 elevation has been shown to contribute to the pathology of cardiovascular diseases, and caveolin-1 peptide was reported to be protective for the heart in myocardial I/R. This effect involved a NO-mediated mechanism.^14^ Caveolin-1 deficiency was shown to aggravate cardiac dysfunction and reduced survival rate in rats that experienced MI.^15^ Although a significant change was not detected in caveolin-levels in our study, other studies are available indicating that myocardial caveolin-1 content decreased following I/R.^14^

In this experimental study, rhokinase levels were detected to increase following I/R. Rhokinase activity has been shown to increase during reperfusion and played an important role in I/R-related myocardial injury.^33^ Animal studies have suggested that rhokinase inhibition protects the heart against I/R injury. Administration of the rhokinase inhibitor, Y-27632, significantly inhibited rhokinase activation in I/R and reduced the infarct area.^33^ In the present study, rhokinase activity was observed to decrease when rosuvastatin was administered. Similarly, rhokinase activity could be inhibited in long-term administration of rosuvastatin and in cell cultures.^34^-^36^.

NFkB is a redox-sensitive transcription factor that is activated in response to oxidative stress and is responsible for the production of inflammatory genes. Reduction in sensitivity to I/R injury in NFkB knock-out mice suggested that NFkB-mediated inflammatory responses play an important role in injury.^37^ The area of the myocardial infarct induced by reperfusion decreased significantly when NFkB activation was blocked through PS-519.^38^ Results of the study showed that reperfusion injury may be inhibited when NFkB activation is suppressed. In the present study, NFkB levels significantly increased with I/R. This increase was significantly reduced when rosuvastatin was administered, and the levels returned to control values.

## Conclusion

The effect of chronic administration of rosuvastatin on oxidative stress, inflammation and endogenous NO generation in I/R injury has been reported for the first time in our study. Rosuvastatin caused inhibition of I/R-mediated increases in related mediators, although not significantly for ADMA and NADPH oxidase levels. We believe that rosuvastatin may be important in treatment protocols of myocardial I/R due to its positive effects on rhokinase, NADPH oxidase, ADMA, hsp 90 and NFkB levels, although further studies are necessary.
